# High-Resolution PTP1B Inhibition Profiling Combined with HPLC-HRMS-SPE-NMR for Identification of PTP1B Inhibitors from *Miconia albicans*

**DOI:** 10.3390/molecules23071755

**Published:** 2018-07-17

**Authors:** Rita de Cássia Lemos Lima, Kenneth T. Kongstad, Lucília Kato, Marcos José das Silva, Henrik Franzyk, Dan Staerk

**Affiliations:** 1 Department of Drug Design and Pharmacology, Faculty of Health and Medical Sciences, University of Copenhagen, Universitetsparken 2, DK-2100 Copenhagen, Denmark; ritalemosrm@gmail.com (R.d.C.L.L.); kenneth.kongstad@sund.ku.dk (K.T.K.); henrik.franzyk@sund.ku.dk (H.F.); 2 Instituto de Química, Universidade Federal de Goiás, Goiânia 70040-010, Brazil; luciliakato@gmail.com; 3 Instituto de Ciências Biológicas, Universidade Federal de Goiás, Goiânia 70040-010, Brazil; marcos_agrorural@hotmail.com

**Keywords:** *Miconia albicans*, type 2 diabetes, PTP1B, HPLC-HRMS-SPE-NMR

## Abstract

Protein tyrosine phosphatase 1B (PTP1B) is an intracellular enzyme responsible for deactivation of the insulin receptor, and consequently acts as a negative regulator of insulin signal transduction. In recent years, PTP1B has become an important target for controlling insulin resistance and type 2 diabetes. In the present study, the ethyl acetate extract of leaves of *Miconia albicans* (IC_50_ = 4.92 µg/mL) was assessed by high-resolution PTP1B inhibition profiling combined with HPLC-HRMS-SPE-NMR for identification of antidiabetic compounds. This disclosed eleven PTP1B inhibitors, including five polyphenolics: 1-*O*-(*E*)-caffeoyl-4,6-di-*O*-galloyl-β-d-glucopyranose (**2**), myricetin 3-*O*-α-l-rhamnopyranoside (**3**), quercetin 3-*O*-(2″-galloyl)-α-l-rhamnopyranoside (**5**), mearnsetin 3-*O*-α-l-rhamnopyranoside (**6**), and kaempferol 3-*O*-α-l-arabinopyranoside (**8**) as well as eight triterpenoids: maslinic acid (**13**), 3-*epi*-sumaresinolic acid (**14**), sumaresinolic acid (**15**), 3-*O*-*cis*-*p*-coumaroyl maslinic acid (**16**), 3-*O*-*trans*-*p*-coumaroyl maslinic acid (**17**), 3-*O*-*trans*-*p*-coumaroyl 2α-hydroxydulcioic acid (**18**), oleanolic acid (**19**), and ursolic acid (**20**). These results support the use of *M. albicans* as a traditional medicine with antidiabetic properties and its potential as a source of PTP1B inhibitors.

## 1. Introduction

Type 2 diabetes is a metabolic syndrome caused by an unbalanced production and/or action of the hormone insulin. Pancreatic β-cells produce insulin as a response to increased blood glucose after digestion of carbohydrates. When insulin binds to the insulin receptor, several downstream signaling pathways become activated, leading to an uptake of glucose from the bloodstream into the cells [1,2,3]. For type 2 diabetics, cells from liver and muscles display a decreased response to the insulin stimuli, i.e., they are insulin-resistant, and the resulting decreased glucose uptake leads to hyperglycemia. Insulin resistance is caused by malfunctions in signal transduction such as impaired activation of the insulin receptor tyrosine kinase or down-regulation of the insulin-stimulated phosphatidylinositol-3-OH kinase (PI3K) [4,5,6]. Therefore, enzymes involved in this signaling pathway constitute promising targets for managing insulin resistance and for treatment of T2D patients. Protein tyrosine kinase 1B (PTP1B) is an intracellular enzyme involved in the negative regulation of insulin signaling by dephosphorylating the activated insulin receptor, and consequently PTP1B inhibitors promote a prolonged effect of insulin [6]. However, at present, there are no PTP1B inhibitors in clinical use, and PTP1B is therefore an intriguing target in the search for new therapeutic alternatives to the drugs currently available for managing T2D.

*Miconia* is a genus comprising approximately 1000 species of flowering plants belonging to the family Melastomataceae, and it is native to neo-tropical forests and widely spread in warm areas of the American continent [7]. *Miconia albicans* is a shrub commonly found on the Brazilian savannah and in the Brazilian rain forests. Extracts obtained from various *Miconia* species have been reported to exhibit antifungal, analgesic, and antiprotozoal activity as well as α-amylase and α-glucosidase inhibitory activity. Although *M. albicans* is used as a traditional anti-inflammatory medicine in Brazil and for treatment of diabetes in Mexico, the pharmacological properties of this plant species have not yet been investigated in detail. Few studies have reported its antifungal and antibacterial activity as well as its protective effects towards doxorubicin-induced DNA damage, but the individual constituents responsible for these effects have not been identified. The only studies of the phytochemistry of *M. albicans* led to isolation of the triterpenes α-amyrin and β-amyrin, and the steroids β-sitosterol and stigmasterol [8,9,10,11].

Bioassay-guided fractionation is a widely used method for identification of bioactive constituents in crude plant extracts, but it is usually both laborious and time-consuming. Thus, the combined use of high-resolution inhibition profiling (HR-inhibition profiling) that pinpoints individual bioactive constituents and high-performance liquid chromatography—high-resolution mass spectrometry—solid-phase extraction—and nuclear magnetic resonance spectroscopy (HPLC-HRMS-SPE-NMR) that allows structural identification from analytical-scale HPLC analysis, can accelerate the search for bioactive constituents in complex plant extracts. HR-inhibition profiling/HPLC-HRMS-SPE-NMR have already been used for accelerated identification of α-glucosidase inhibitors [12,13], α-amylase inhibitors [14], PTP1B inhibitors [15], monoamine oxidase inhibitors [16], and antioxidants [17,18] directly from crude extracts of foods and herbal medicine. In this study, we report the PTP1B inhibitory activity of crude defatted ethyl acetate extract of *M. albicans* as well as the identification of several active polyphenolics and triterpenoids by the use of high-resolution PTP1B inhibition profiling combined with HPLC-HRMS-SPE-NMR.

## 2. Results

The crude defatted extract of *M. albicans* was found to possess high PTP1B inhibitory activity with an IC_50_ value of 4.92 ± 0.31 μg/mL (as determined from the dose-response curve shown in Supplementary Material Figure S1), and it was therefore decided to identify some of the bioactive constituents responsible for this inhibitory activity.

### 2.1. High-Resolution PTP1B Inhibition Profiling and Identification of Active Compounds from Crude Extract of M. albicans

**Figure 1:**
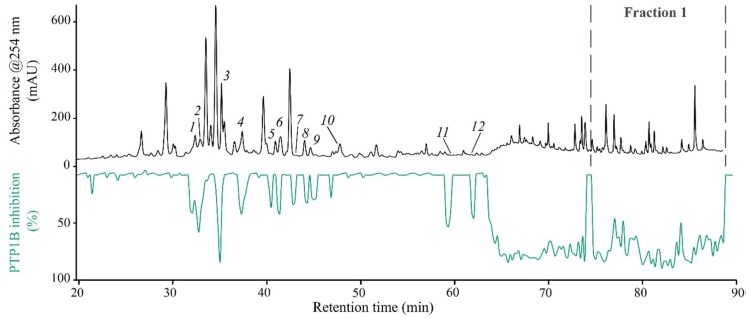
High-resolution PTP1B inhibition profiling of the crude ethyl acetate extract of *M. albicans*. Black trace: UV absorption at 254 nm; Green trace: PTP1B inhibition profiling.

The crude extract was subjected to high-resolution PTP1B inhibition profiling, and the biochromatogram (Figure 1) displayed 12 distinct peaks corresponding to moderate to strong activity eluting between 32 and 62 min. In addition, two large humps with around 100% inhibition were observed in the retention ranges 64–75 min and 75–90 min. Initially, HPLC-HRMS-SPE-NMR analysis of crude extract was performed to identify the material eluted with HPLC peaks *1–12*, which are correlated with PTP1B inhibitory activity. HRMS and NMR data are reported in Supplementary Material Table S1.

The material eluting with peak *1* showed a molecular ion with *m*/*z* 615.0997 [M − H]^−^ suggesting the presence of a compound with molecular formula C_28_H_24_O_16_ (ΔM = −0.8 ppm), but the amount and purity of the material did not allow for further structural information based on NMR. 

**Figure 2:**
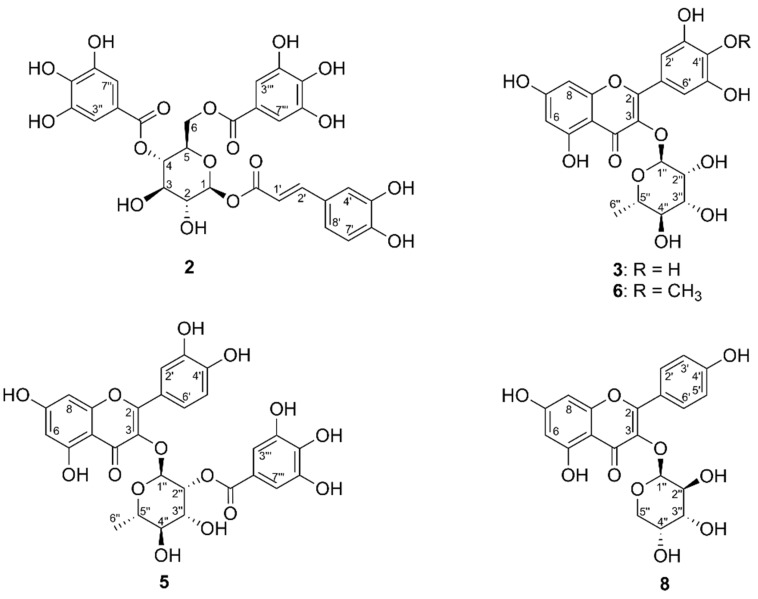
PTP1B inhibitory polyphenolics from extract of *M. albicans.*

The compound eluting with peak *2* showed a molecular ion with *m*/*z* 647.1214 [M + H]^+^ suggesting a molecular formula of C_29_H_26_O_17_ (ΔM = 4.4 ppm). The ^1^H NMR spectrum showed characteristic signals for a caffeoyl group (δ 7.58, 1H, d, 16.0 Hz, H-2′; δ 7.77, 1H, d, 2.1. Hz, H-4′; δ 7.55, 1H, dd, 8.3, 2.1 Hz, H-8′; δ 7.06, 1H, d, 8.3 Hz, H-7′; δ 6.28, 1H, d, 16.0 Hz, H-1′), two galloyl groups (δ 6.97 and 6.90, s, 2H each, H-3″/H-7″ and H-3‴/H-7‴), and a 1,4,6-triacylated glucose moiety (δ 5.10, 1H, d, 8.0 Hz, H-1; δ 4.56, 1H, m, H-4; δ 4.46, 1H, dd, 11.3, 7.6 Hz, H-6B; δ 4.33, 1H, dd, 11.3, 7.0 Hz, H-6A). Comparison with ^1^H NMR data from literature led to identification of **2** as 1-*O*-(*E*)-caffeoyl-4,6-di-*O*-galloyl-β-d-glucopyranose [19] (Figure 2).

The material eluting with peaks *3* and *4* showed molecular ions with *m*/*z* 463.0880 [M − H]^−^ and *m*/*z* 599.1047 [M − H]^−^, suggesting molecular formulas of C_21_H_20_O_12_ (ΔM = 0.4 ppm) and C_28_H_24_O_15_ (ΔM = −0.8 ppm), respectively. The ^1^H NMR spectrum of **3** showed signals characteristic for myricetin (δ 6.95, 2H, s, H-2′/H-6′; δ 6.37, 1H, d, 2.2 Hz, H-8; δ 6.20, 1H, d, 2.2 Hz, H-6), and a rhamnose unit (δ 5.32, 1H, d, 1.5 Hz, H-1”; δ 4.22, 1H, dd, 3.4, 1.7 Hz, H-2”; δ 0.94, 3H, d, 6.8 Hz, H-6″). Comparison with literature data allowed identification of **3** as myricetin 3-*O*-α-l-rhamnopyranoside [20]. The molecular formula of **4** might indicate this compound to be a galloylated quercetin rhamnoside. However, the amount and purity of the material eluted with peak *4* did not allow further structural information based on NMR spectroscopy.

The compound eluting as peak *5* showed a molecular ion of *m*/*z* 599.1041 [M − H]^−^ suggesting the molecular formula C_28_H_24_O_15_ (ΔM = 0.2 ppm). The ^1^H NMR spectrum displayed signals characteristic for quercetin (δ 7.34, 1H, d, 2.1 Hz, H-2′; δ 7.30, 1H, dd, 8.5, 2.1 Hz, H-6′; δ 2.92, 1H, d, 8.5 Hz, H-5′; δ 6.38, 1H, d, 2.1 Hz, H-8; δ 6.21, 1H, d, 2.1 Hz, H-6), a galloyl unit (δ 6.89, 2H, s), and a rhamnose unit (δ 5.34, 1H, d, 1.8 Hz, H-1″; δ 4.78, 1H, dd, 3.4, 1.7 Hz, H-2″; δ 3.75, 1H, dd, 9.5, 3.4 Hz, H-3″; δ 3.34, m, H-4″; δ 3.42, 1H, dd, 9.3, 6.2 Hz, H-5″; δ 0.94, 3H, d, 6.8 Hz, H-6″). This indicated **5** to be a galloylated quercetin rhamnoside, and the downfield shift of H-2″ (δ 4.78) inferred the galloyl unit to be attached in position 2″. Comparison with data from literature allowed identification of **5** as quercetin 3-*O*-(2″-galloyl)-α-l-rhamnopyranoside [21].

The compound eluting with peak *6* exhibited a molecular ion with *m*/*z* 477.1041 [M − H]^−^ suggesting the molecular formula C_20_H_22_O_12_ (ΔM = −0.5 ppm). The ^1^H NMR spectrum showed a singlet at δ 6.88 (2H, H-2″/H-6″) together with two doublets at δ 6.38 and 6.22, each integrating to 1H (*J*_H-6,H-8_ = 2.0 Hz) corresponding to H-6 and H-8 being *meta*-positioned. An additional 3H singlet at δ 3.88 indicated the presence of a methoxy group, and HMBC correlations showed it to be attached to C-4′. A doublet at δ 5.31 ppm (1H, *J*_H-1″,H-2″_ = 1.8 Hz) together with a doublet at δ 0.94 (3H, *J*_H-6″,H-5″_ = 6.8 Hz, H-6″) and signals in the region δ 3.33–4.25 suggested the presence of a rhamnose moiety. Comparison of these data with literature allowed identification of **6** as mearnsetin 3-*O*-α-l-rhamnopyranoside [22].

The material eluting with peak *8* showed a molecular ion with *m*/*z* 417.0825 [M − H]^−^ suggesting the molecular formula C_20_H_18_O_10_ (ΔM = 0.5 ppm). The ^1^H NMR spectrum contained signals corresponding to kaempferol (δ 8.06 and 6.89, 2H each, AA’ and XX’ systems; δ 6.41, 1H, d, 2.1 Hz, H-8; δ 6.21, 1H, d, 2.1 Hz, H-6) and an arabinose unit (δ 5.14, 1H, d, 6.4 Hz, H-1″ and signals from δ 3.15–3.80, 5H). Comparison with ^1^H NMR data from literature allowed identification of **8** as kaempferol 3-*O*-α-l-arabinopyranoside [23] (Figure 2).

The HRMS data and the inferred molecular formula of the compounds eluting with peaks *7* and *9–12* are given in Table S1 in Supplementary Data, but the amount and purity of these compounds did not allow further structural identification.

Dereplication of the hump from 64 to 75 min correlated with PTP1B inhibitory activity, and based on LC-ESI-HRMS/MS data it showed losses of [M − H − 44]^−^ and [M − H − 62]^−^, corresponding to a loss of CO_2_ and CO_2_ + H_2_O, which is common for lipids (fatty acids). This suggested that this area contained large amounts of structurally related lipids. Two previous studies have shown that some classes of lipids possess PTP1B inhibitory activity [24,25], which could support the activity observed in the biochromatogram, but the amount and purity of material obtained from this region did not allow structural identification of individual metabolites.

HRMS-based dereplication of peaks eluting from 75 to 90 min suggested the presence of several structurally related pentacyclic triterpenes and saponins. There is a growing interest in naturally occurring triterpenoids because they exhibit a wide spectrum of biological activities including antibacterial, fungicidal, antiviral, cytotoxic, anticancer, and cardiovascular activity. In addition to this, some triterpenoids inhibit PTP1B, a key regulatory enzyme for regulation of blood glucose [26,27,28,29,30]. The fraction eluting from 75 to 90 min was therefore isolated by preparative-scale HPLC (hereafter called fraction **F1**) to allow further analysis using high-resolution PTP1B inhibition profiling combined with HPLC-HRMS-SPE-NMR.

### 2.2. High-Resolution PTP1B Inhibition Profiling and Identification of Active Compounds from **F1**

**Figure 3:**
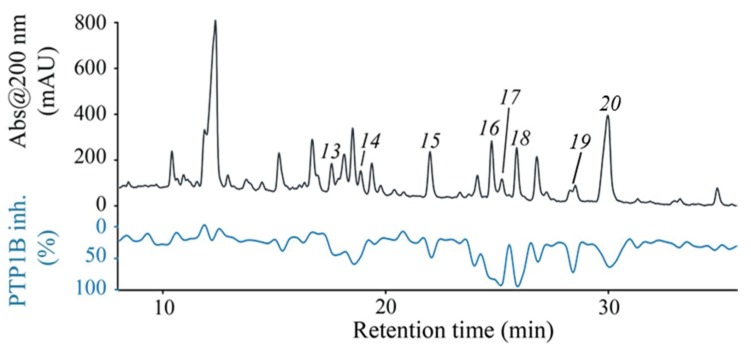
High-resolution PTP1B inhibition profiling of fraction **F1**. Black trace: UV absorption at 200 nm; Green trace: PTP1B inhibition profiling.

High-resolution PTP1B inhibition profiling of the triterpenoid-containing fraction **F1** obtained by preparative-scale HPLC provided the biochromatogram shown in Figure 3. This displayed several HPLC peaks correlated with moderate to strong PTP1B-inhibitory activity—varying from 33% to 95% inhibition. Initial dereplication of the chromatographic peaks eluted from fraction **F1** indicated, based on HRMS and UV data, the presence of several structural isomers of triterpenoids. This fraction was therefore further analyzed by HPLC-HRMS-SPE-NMR to enable identification of PTP1B-inhibitory triterpenoids. Thus, peaks *13–20* were cumulatively trapped (from 10 consecutive separations) on Hysphere GP-resin cartridges based on threshold levels from the UV chromatogram (254 or 280 nm), total ion chromatogram or base peak chromatogram. After drying with nitrogen gas, the trapped metabolites were eluted into 1.7-mm NMR tubes, and 600 MHz spectra were acquired. 

**Figure 4:**
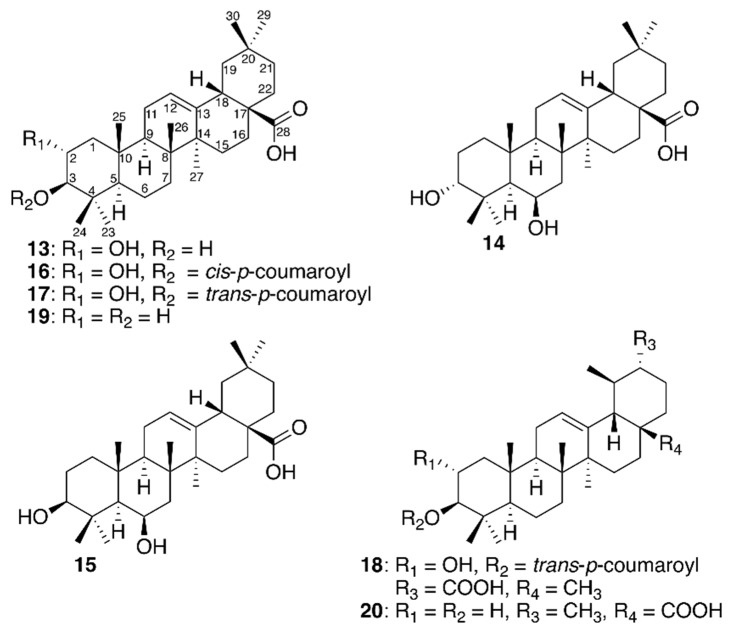
PTP1B inhibitors identified from fraction **F1** of *M. albicans*.

The compound obtained by repeated trapping of peak *13* showed a molecular ion with *m*/*z* 473.3620 [M + H]^+^ suggesting the molecular formula C_30_H_48_O_4_ (ΔM = 0.5 ppm). The ^1^H NMR spectrum revealed the presence of signals characteristic of an olean-12-en-oic acid: a single olefinic proton (δ 5.27, t, 3.6 Hz, H-12), seven methyl groups: δ 1.00 (CH_3_-23), δ 0.81 (CH_3_-24), δ 1.01 (CH_3_-25), δ 0.82 (CH_3_-26), δ 1.16 (CH_3_-27), δ 0.91 (CH_3_-29), and δ 0.94 (CH_3_-30), a triplet of doublets at δ 3.62 (1H, 9.5 and 4.1 Hz, H-2) and a doublet at δ 2.91 (9.5 Hz, H-3) indicating two trans-diaxial protons both connected to oxygenated carbons. Comparison with ^1^H NMR data from literature confirmed **13** to be maslinic acid [31] (Figure 4). 

The compound obtained by repeated trapping of peak *14* showed a molecular ion with *m*/*z* 473.3625 [M + H]^+^ suggesting the molecular formula C_30_H_48_O_4_ (ΔM = 1.7 ppm). The ^1^H NMR spectrum displayed signals similar to those for compound **13**, indicating that **14** was an olean-12-en-oic acid as well. The presence of signals at δ 1.48 (1H, dq, *J*_H2eq,H2ax_ = 14.4 and *J*_H2eq,H1eq_ = *J*_H2eq,H1ax_ = *J*_H2eq,H3eq_ = 3.2 Hz, H-2α), δ 2.03 (1H, dddd, *J*_H2ax,H2eq_ = 14.4, *J*_H2ax,H1ax_ = 9.5, *J*_H2ax,H1eq_ = 3.3, *J*_H2ax,H3eq_ = 2.8 Hz, H-2β), and a triplet at δ 3.28 (1H, *J*_H3eq,H2ax_ = *J*_H3eq,H2eq_ = 2.8 Hz, H-3) show that C-3 has a β-positioned equatorial hydrogen and an α-positioned axial hydroxy group. The signal for H-6 at δ 4.37 is a broad singlet, with only equatorial-equatorial and equatorial-axial couplings, showing a β-positioned axial hydroxy group. Analysis of COSY, NOESY, HSQC and HMBC spectra as well as comparison with ^1^H and ^13^C NMR data from literature of the related methyl ester [32] allowed identification of **14** as 3-*epi*-sumaresinolic acid (Figure 4). 

The compound obtained by repeated trapping of peak *15* showed also a molecular ion with *m*/*z* 473.3618 [M + H]^+^ suggesting the same molecular formula C_30_H_48_O_4_ (ΔM = 1.5 ppm). The ^1^H NMR spectrum displayed signals similar to those for compound **14**, but with H-3 appearing at δ 3.06 as a doublet of doublet with *J*_H3ax,H2ax_ = 12 Hz and *J*_H3ax,H2eq_ = 4.1 Hz. Analysis of COSY, NOESY, HSQC and HMBC spectra as well as comparison with ^1^H and ^13^C NMR data from literature of the related methyl ester [32] allowed identification of **15** as sumaresinolic acid (Figure 4).

The compounds obtained by repeated trapping of peaks *16* and *17* showed molecular ions with *m*/*z* 619.3979 [M + H]^+^ and *m*/*z* 619.3989 [M + H]^+^, suggesting molecular formulas of C_39_H_54_O_6_ for both **16** and **17** (ΔM = 2.7 and 0.7 ppm, respectively). The ^1^H NMR spectra resembled that of **13** but revealed additional signals for an AA’XX’ aromatic system as well as two *cis*-positioned olefinic protons (δ 6.88 and δ 5.85, 1H each, 12.8 Hz) for **16** and two *trans*-positioned olefinic protons (δ 7.64 and δ 6.81, 1H each, 15.9 Hz) for **17**. This indicated **16** and **17** to be *cis*- and *trans*-coumaroyl esters of **13**. A clear downfield shift of H-3 from δ 2.91 in **13** to δ 4.63 in **16** and δ 4.64 in **17**, shows the coumaroyl unit to be attached at C-3 in both compounds. By comparison of these ^1^H NMR data with literature data the structure of **16** and **17** were assigned as 3-*O*-*cis*-*p*-coumaroyl-maslinic acid and 3-*O*-*trans*-*p*-coumaroyl-maslinic acid, respectively [33]. The compound obtained by repeated trapping of peak *18* showed also a molecular ion of *m*/*z* 619.3974 [M + H]^+^ suggesting the same molecular formula C_39_H_54_O_6_ (ΔM = 3.1 ppm) as that of **16** and **17**. The ^1^H NMR spectrum showed signals characteristic of an aromatic AA’XX’ system and two *trans*-coupled olefinic protons (δ 7.63 and 6.38, 1H each, 15.8 Hz) inferring the presence of a *trans*-coumaroyl unit. Contrary to the seven methyl singlets observed for **13**–**17**, the ^1^H NMR spectrum of **18** showed six methyl singlets and a methyl doublet attributed to a methyl group at C-19 (δ 0.95, 3H, d, 6.1 Hz, CH_3_-29). Further analysis based on 1D and 2D NMR and comparison with literature data established that **18** was 3-*O*-*trans*-*p*-coumaroyl-2α-hydroxydulcioic acid [34] (Figure 4). 

The compound obtained by repeated trapping of peak *19* showed a molecular ion with *m/z* 457.3668 [M + H]^+^ suggesting the molecular formula C_30_H_48_O_3_ (ΔM = 1.8 ppm). Similar to the spectra of **13**–**15**, the ^1^H NMR spectrum of **19** displayed signals characteristic of an olean-12-oic acid, and comparison with NMR data from literature allowed identification of **19** as oleanolic acid [35] (Figure 4). The compounds obtained by repeated trapping of peak *20* showed a molecular ion with *m/z* 457.3672 [M + H]^+^ suggesting the molecular formula C_30_H_48_O_3_ (ΔM = 0.9 ppm). The ^1^HNMR spectrum of **20**, showed signals characteristic of an ursan-12-oic acid, and comparison with NMR data from literature allowed identification of **20** as ursolic acid [36]. NMR data of **13**–**20** are given in Supplementary Material (Table S1).

Dose-response curves of **13**–**20** are shown in Supplementary Material (Figure S2), and the derived IC_50_ values are presented in Table 1. Maslinic acid has previously been reported as a bioactive pentacyclic triterpene acid, with anti-tumor, antioxidant, anti-HIV, and antimicrobial activity. In this study **13** was identified as a PTP1B inhibitor with an IC_50_-value of 3.21 ± 0.8 μM, which is in agreement with the previously reported IC_50_-value of 5.93 μM [29]. Moreover, studies suggest that PTP1B inhibition of this type of pentacyclic triterpenes is affected by the configuration and substituents at C-2 and C-3 [30]. Although the biochromatogram does not give quantitative pharmacological responses, Figure 3 shows that compounds **16** (IC_50_: 0.46 ± 0.07 μM), **17** (IC_50_: 1.08 ± 0.32 μM), and **18** (IC_50_: 1.6 ± 0.50 μM) with a coumaroyl unit at C-3 are correlated with higher PTP1B inhibitory activity as compared to that of **13**. According to Qiu and colleagues [30], this structural feature may not only increase the potency but also improve the selectivity of these compounds for PTP1B. PTP1B belongs to the protein tyrosine kinases (PTPs), a family comprising approximately 100 enzymes that are involved in vital processes in the human body, and it has therefore been a challenge to identify selective PT1B inhibitors that might be developed into new medicines [6,30,37]. The present work demonstrates that *M. albicans* constitute a rich source of new potent 3-coumaroyl analogues of maslinic acid, and presents the PTP1B inhibitory activity of **14**–1**8** for the first time. However, further studies are needed to investigate their potential as selective PTP1B inhibitors.

## 3. Materials and Methods 

### 3.1. Chemicals and Reagents

*p*-Nitrophenyl phosphate (*p*NPP), dimethyl sulfoxide (DMSO), sodium phosphate monobasic dihydrate, sodium phosphate dibasic, sodium hydroxide, sodium azide, tris-(hydroxymethyl)-aminomethane (Tris), bis-(2-hydroxyethyl)-imino-tris-(hydroxymethylmethane) (Bis-Tris), *N*,*N*,*N*′,*N*′-ethylenediaminetetraacetate (EDTA), dithiothreitol (DTT), methanol-*d*_4_, and HPLC grade acetonitrile were purchased from Sigma-Aldrich (St. Louis, MO, USA), recombinant human PTP1B (BML-SE332-0050, EC 3.1.3.48) was purchased from Enzo Life Sciences Inc. (Farmingdale, NY, USA), and formic acid was purchased from Merck (Darmstadt, Germany). Water was purified by deionization and filtration through a 0.22 μm membrane (Millipore, Billerica, MA, USA).

### 3.2. Preparation of Crude Ethyl Acetate Extracts of Leaves of M. albicans

Leaves of *Miconia albicans* were collected in the National Park of Chapada dos Veadeiros (Alto Paraíso de Goiás, GO, Brazil) in February 2016, identified by botanist Dr. Marcos José da Silva (Universidade Federal de Goiás), and a voucher specimen was deposited at the herbarium of Universidade Federal de Goiás with accession number UFG 7163. The leaves were dried and powdered, and a batch (139.2 g) of this plant material was extracted twice with ethyl acetate (1.0 L) for 2 h under sonication. The extract was filtered and concentrated under reduced pressure to afford a crude extract (9.0 g). Fatty acids and chlorophylls were removed by liquid-liquid partitioning between 90% (*v*/*v*) methanol (0.1 L) and petroleum ether (0.9 L), yielding a crude defatted extract (4.0 g) after evaporation of the methanol fraction under reduced pressure.

### 3.3. Microplate-Based PTP1B Inhibition Assay

PTP1B inhibition assaying of the defatted extract, fraction **F1** (from preparative HPLC: see Section 3.5) and microfractions (obtained as described below in Section 3.4) were performed according to the microplate-based method previously described [38]. In brief, all solutions were prepared in a buffer of 50 mM Tris and 50 mM Bis-Tris containing 100 mM NaCL and adjusted to pH 7. To each well containing microfractions or dilution series of pure compounds were added 18 µL of DMSO and 52 µL of 3.4 mM EDTA in Bis-Tris buffer (to give final well concentrations of 10% DMSO and 1 mM EDTA) as well as 60 µL of a Bis-Tris buffer solution containing 1.5 mM *p*NPP and 6 mM DTT in phosphate buffer (to give final well concentrations of 0.5 mM *p*NPP and 2 mM DTT). The microplate was incubated for 10 min at 25 °C, and the reaction was started by addition of 0.001 μg/μL PTP1B stock solution (50 μL) in phosphate buffer (to give a final concentration of 0.05 µg PTP1B per well). The reaction was monitored at 405 nm every 30 s for 10 min to yield the enzyme activity (expressed as cleavage rate AU/s) by using a Thermo Scientific Multiskan FC microplate photometer (Thermo Scientific, Waltham, MA, USA). For each microplate, blank samples (negative controls) containing buffer, enzyme, EDTA, DTT, and *p*NPP were measured in triplicate, and for the dilution series, RK682 was included as a reference inhibitory compound (i.e., positive control). The percentage inhibition of PTP1B was calculated according to the following equation:

$$\%\;\mathrm{inhibition}=\left(1-\frac{{\mathrm{SLOPE}}_{\mathrm{sample}}}{{\mathrm{SLOPE}}_{\mathrm{blank}}}\right)\times 100$$

The high-resolution PTP1B biochromatograms (high-resolution PTP1B inhibition profiles) were constructed by plotting the inhibition values against the chromatographic retention time. Dose-response curves and IC_50_ values for dilution series of the crude extract and pure compounds were obtained using GraFit software, version 5.0.11 (Erithacus Software Limited, West Sussex, UK). For PTP1B IC_50_ values, the crude extract and isolated compounds were assessed by using the standard assay conditions described above. Nine dilutions were assessed in triplicates and percentage inhibition calculated as mean ± standard deviation in Microsoft Excel according to the above equation. The results were imported and used for assessing dose-response curves and IC_50_ values in GraFit software, version 5.0.11 (Erithacus Software Limited). Data were fitted to the equation

$$f\left(x\right)=\min+\frac{\max-\min}{1+{\left(\frac{x}{{\mathrm{IC}}_{50}}\right)}^{\mathrm{slope}}}$$

where min is the background, max–min is the y-range, x is the concentration, and slope is the Hill slope. Results are reported as IC_50_ values ± standard error.

### 3.4. Microfractionation

Microfractionation of the extract of *M. albicans* for high-resolution PTP1B inhibition profiling was conducted using an Agilent 1200 system (Santa Clara, CA, USA—HPLC system 1) comprising a G1311A quaternary pump, a G1322A degasser, a G1316A thermostatted column compartment, a G1315C photodiode array detector, a G1367C high-performance autosampler, and a G1364C fraction collector, controlled by Agilent ChemStation version B.03.02 software. Separations were performed by using a reversed-phase Phenomenex Luna C_18_(2) column (150 mm × 4.6 mm i.d., 3 μm particle size, 100 Å pore size) (Phenomenex, Torrance, CA, USA). A binary elution system of water: acetonitrile (95:5, solvent A) and acetonitrile: water (95:5, solvent B), both acidified with 0.1% formic acid, was used with a flow rate of 0.8 mL/min. An aliquot (10 μL) of a solution of the crude extract (equivalent to 200 μg of crude extract) was chromatographically separated using the following stepwise linear gradient elution profile: 0 min, 0% B; 50 min, 20% B; 60 min, 30% B; 65 min, 50% B; 85 min, 100% B; 105 min, 100% B. The chromatographic eluate was collected into four 96-well microplates (308 wells) from 20 to 90 min, leading to a biochromatogram with a resolution of 4.4 data points/min. The HPLC separation of fraction **F1** was conducted using a flow rate of 0.8 mL/min, and a binary elution gradient of water:acetonitrile (95:5, solvent A) and acetonitrile:water (95:5, solvent B), both acidified with 0.1% formic acid, and the following stepwise linear elution gradient: 0 min, 50% B; 5 min, 55% B; 35 min, 57% B; 36 min, 70% B; 40 min, 90% B; 41 min, 100% B; 51 min, 100% B. For the high-resolution inhibition profiling of fraction **F1**, the HPLC eluates in the range 8 min to 36 min were collected into one 96-well microplate (88 wells used) leading to a resolution of 3.1 data points/min.

### 3.5. Preparative-Scale HPLC for Fractionation of the Crude Extract of M. albicans

A solution of crude extract of *M. albicans* was prepared in methanol (100 mg/mL) and fractionated by 10 consecutive injections of 500 μL using an Agilent 1100 series HPLC system (Santa Clara, CA, USA—HPLC system 2) comprising a G1361A quaternary pump, a G2260A autosampler, and a G1365B MWD detector, all controlled by Agilent ChemStation version 3.02 software, equipped with a Phenomenex (Phenomenex Inc., Torrance, CA, USA) Luna C_18_(2) column (250 mm × 21.2 mm i.d., 5 μm particle size, 100 Å pore size). A binary elution system of water: acetonitrile (95:5, solvent A) and acetonitrile:water (95:5, solvent B), both acidified with 0.1% formic acid was employed under flow rate of 17 mL/min. The stepwise linear elution gradient was as follows: 0 min, 0% B; 50 min, 20% B; 60 min, 30% B; 65 min, 50% B; 85 min, 100% B; 105 min, 100% B. Fraction **F1** was collected from 74 to 90 min (82.0 mg).

### 3.6. Isolation of PTP1B Inhibitors from the Extract of M. albicans by Analytical-Scale HPLC

The early-eluting PTP1B inhibitors (observed from 32 to 60 min) were isolated directly from the crude extract using HPLC system 1 operated with the same column, solvent system and flow rate as described in Section 3.4. Twenty successive injections of 20 μL of a solution of the crude extract (equivalent to 400 μg of crude extract each) were separated using the stepwise linear elution gradient: 0 min, 0% B; 50 min, 20% B; 60 min, 30% B; 65 min, 50% B; 85 min, 100% B; 105 min, 100% B. The active compounds observed in the inhibition profile were collected, dried and submitted to analysis by NMR spectroscopy.

### 3.7. HPLC-HRMS-SPE-NMR Analyses of Triterpenoids from **F1**

HPLC-HRMS-SPE-NMR analyses of fraction **F1** were performed on a system comprising an Agilent 1260 HPLC (Santa Clara, CA, USA), a Bruker micrOTOF-Q II mass spectrometer (Bruker Daltonik, Bremen, Germany), a Knauer Smartline K120 pump (Knauer, Berlin, Germany), a Spark Holland Prospekt-2 SPE unit (Spark Holland, Emmen, The Netherlands), a Gilson 215 liquid handler (Gilson, Middleton, WI, USA), and a Bruker Avance III 600 MHz NMR spectrometer (Bruker Biospin, Rheinstetten, Germany). The Agilent HPLC system consisted of a G1329B autosampler, a G1311B quaternary pump with built-in degasser, a G1316A thermostatted column compartment, equipped with Phenomenex (Phenomenex Inc., Torrance, CA, USA) Luna C_18_(2) column (150 mm × 4.6 mm i.d., 3 μm particle size, 100 Å pore size), and a G1315D photodiode array detector, and the column, elution solvents, temperature, and flow rate were the same as described in Section 3.4. A T-piece after the HPLC column directed approximately 1% of the eluate to the micrOTOF-Q II mass spectrometer equipped with an electrospray ionization source. Spectra were acquired in positive ionization mode, using a drying temperature of 200 °C, a capillary voltage of 4100 V, a nebulizer pressure of 2.0 bar, and a drying gas flow of 7 L/min. To enable internal mass calibration, a solution of sodium formate clusters was automatically injected at the beginning of each run. The remaining 99% of the eluate was directed to the photodiode array detector and subsequently diluted with water at a flow rate of 1 mL/min delivered by the Knauer pump. Hysphere GP-resin SPE cartridges (10 × 2 mm i.d., Spark Holland, Emmen, The Netherlands) were preconditioned with 500 μL of acetonitrile and equilibrated with 500 μL of water. Selected chromatographic peaks were trapped from 10 consecutive chromatographic separations using threshold levels from the UV chromatogram (200 nm and 254 nm), the total ion chromatograms (TIC), or base peak chromatograms (BPC) to trigger trapping. The loaded SPE cartridges were dried with pressurized N_2_ gas for 30 min and subsequently eluted with methanol-d_4_ (30 μL) into 1.7-mm o.d. NMR tubes (Bruker Biospin, Rheinstetten, Germany) using the Gilson liquid handler equipped with a 1-mm needle. HPLC separations, mass spectrometry and analyte trapping on SPE cartridges were controlled by Hystar version 3.2 software (Bruker Daltonik, Bremen, Germany), whereas the elution processes were controlled by Prep Gilson ST version 1.2 software (Bruker Biospin, Rheinstetten, Germany).

Ten successive injections of 12 μL of methanolic solution of **F1** (equivalent to 720 μg) were separated using a binary elution system consisted of water:acetonitrile (95:5, solvent A) and acetonitrile:water (95:5, solvent B), both acidified with 0.1% formic acid, under a flow rate of 0.8 mL/min. The separation was performed using the following stepwise linear gradient elution: 0 min, 50% B; 55 min, 80% B; 60 min, 100% B; 62 min, 100% B.

### 3.8. NMR Experiments

NMR experiments were recorded at 300 K in methanol-d_4_ and acquired on a Bruker Avance III 600 MHz NMR spectrometer (^1^H operating frequency of 600.13 MHz) equipped with a Bruker SampleJet sample changer and a 1.7-mm cryogenically cooled inverse triple-resonance TCI probe (Bruker Biospin, Rheinstetten, Germany). The one-dimensional ^1^H spectra were acquired with 30° pulses with a 20 ppm spectral width, 2.72 s acquisition time, and 1.0 s relaxation delay, collecting 256 or 512 FIDS, each consisting of 64 k data points, and Fourier transformed to 256 k data points with a line broadening of 0.1 Hz. Two-dimensional homonuclear DQF-COSY and NOESY experiments were recorded using a gradient-based pulse sequence with a 20 ppm spectral width and 2 k × 512 data points (processed with forward linear prediction to 1 k data points). Multiplicity-edited HSQC spectra were acquired with 12 ppm spectral width for ^1^H and 170 ppm for ^13^C, 2 k × 256 data points, and 1.0 s relaxation delay. HMBC experiments were recorded with a 12 ppm spectral width for ^1^H and 210 ppm for ^13^C, 2 k × 192 data points and 1.0 s relaxation delay.

## 4. Conclusions

In the present study, five flavonoids and eight triterpenoid PTP1B inhibitors were identified by analysis of the defatted ethyl acetate extract of *M. albicans*. These included maslinic acid derivatives with substituents at position C-3, which seem to be potential selective inhibitors. Thus, the combination of high-resolution inhibition profiling and HPLC-HRMS-SPE-NMR proved to be a powerful tool for accelerated assessment of plant extracts by providing high-quality analytical data. Further studies to explore the selectivity of the 3-coumaroylated maslinic acid are needed.

## Figures and Tables

**Table 1:** IC_50_ values of compounds **13**–**20** isolated from leaves of *Miconia albicans*.

Compound	Name	IC_50_ (μM) *^a^*
**13**	Maslinic acid	3.21 ± 0.8
**14**	3-*epi*-sumaresinolic acid	2.87 ± 0.4
**15**	Sumaresinolic acid	1.84 ± 0.3
**16**	3-*O*-*cis*-*p*-coumaroyl-maslinic acid	0.46 ± 0.07
**17**	3-*O*-*trans*-*p*-coumaroyl-maslinic acid	1.08 ± 0.3
**18**	3-*O*-trans-*p*-coumaroyl-2α-hydroxydulcioic acid	1.6 ± 0.5
**19**	Oleanolic acid	2.88 ± 0.6
**20**	Ursolic acid	2.18 ± 0.8

*^a^ n* = 3.

## Supplementary Materials

The following material is available online: Figures S1 and S2 with dose-response curves for crude ethyl acetate extract and compounds **13**–**20** and Table S1 with HRMS and/or ^1^H NMR data acquired in the HPLC-HRMS-SPE-NMR mode of the material eluted with peaks 1–20 of *Miconia albicans*.
