# Supplementary material for: High-Resolution PTP1B Inhibition Profiling Combined with HPLC-HRMS-SPE-NMR for Identification of PTP1B Inhibitors from Miconia albicans
Source: Molecules. 2018 Jul 17;23(7):1755. doi: 10.3390/molecules23071755 (PMC6100288; doi:10.3390/molecules23071755)
Supplement: Supplementary file 1 [file molecules-23-01755-s001.pdf]

Supplementary material for

High-resolution PTP1B inhibition profiling combined with HPLC-HRMS-SPE-NMR for identification of PTP1B inhibitors from *Miconia albicans*

Rita de Cássia L. Lima[1], Kenneth T. Kongstad[1], Lucília Kato[2], Marcos José das Silva[3], Henrik Franzyk[1] and Dan Staerk[1]*

[1]Department of Drug Design and Pharmacology, Faculty of Health and Medical Sciences, University of Copenhagen, Universitetsparken 2, DK-2100, Copenhagen, Denmark; rita.lima@sund.ku.dk (R.C.L.L.); kenneth.kongstad@sund.ku.dk (K.T.K.); hf@sund.ku.dk (H.F.); ds@sund.ku.dk (D.S.)

[2]Instituto de Química, Universidade Federal de Goiás, Goiânia-GO, Brazil; luciliakato@gmail.com

[3]Instituto de Ciências Biológicas, Universidade Federal de Goiás, Goiânia-GO, Brazil; marcos_agrorural@hotmail.com

* Correspondence: ds@sund.ku.dk; Tel.: +45-3533-6177

Figure S1. Dose-response curve of the defatted ethyl acetate extract of leaves of *Miconia albicans*.

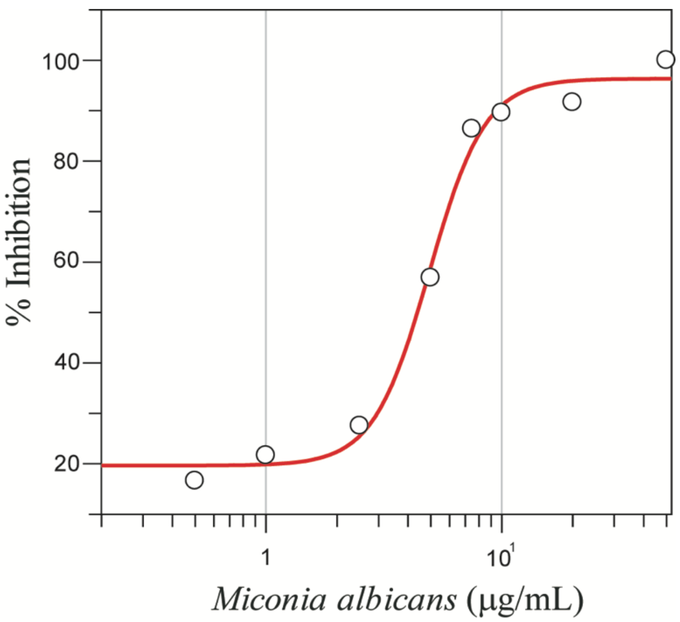

Figure S2. Dose-response curves of compounds **13-20** isolated from leaves of *Miconia albicans*.

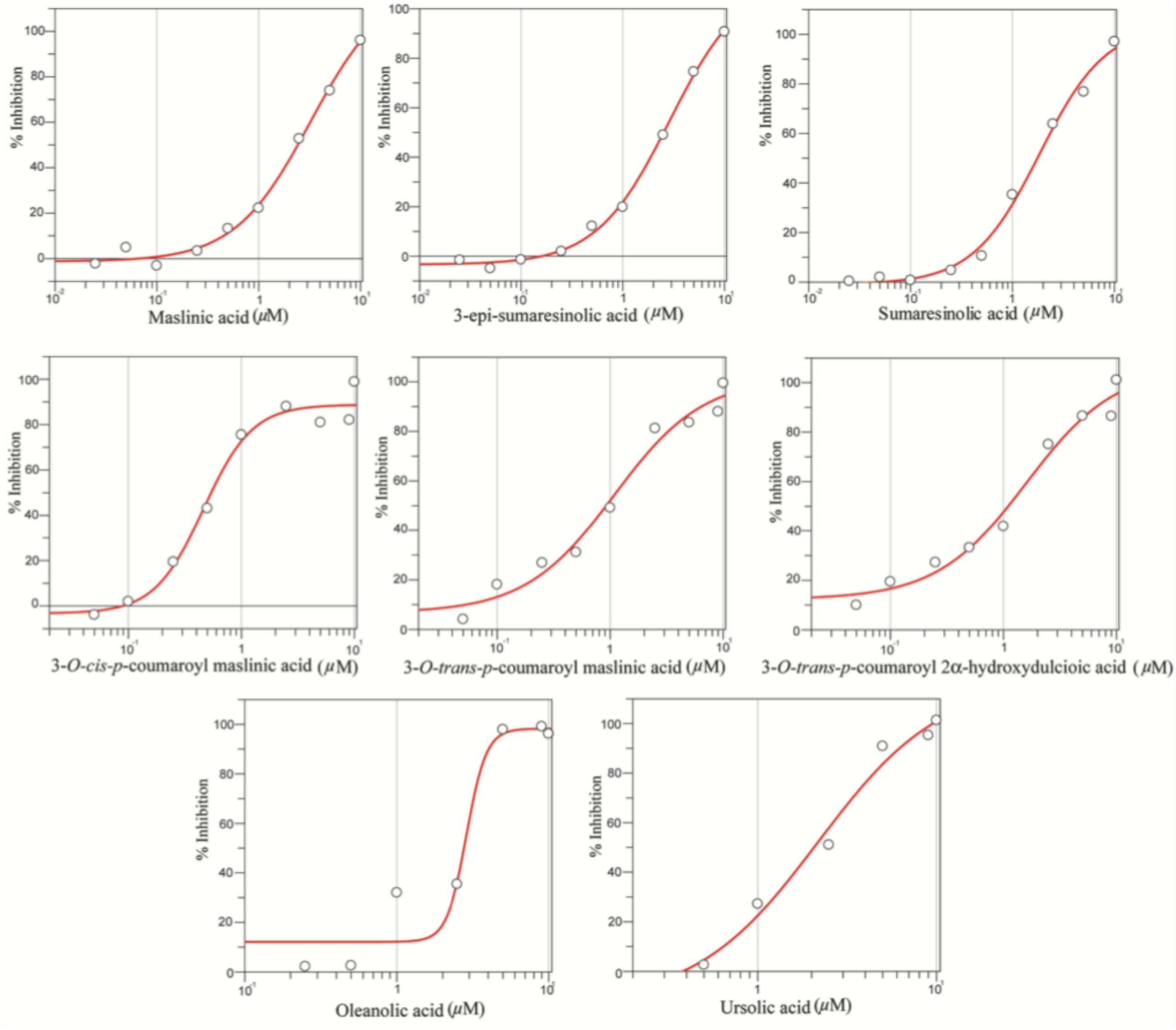

Table S1. Peak number, name, HRMS, ^{1}H NMR and ^{13}C NMR data for PTP1B inhibitors from *Miconia albicans* acquired in the HPLC-HRMS-SPE-NMR mode

Peak	Name	HRMS (molecular formula, Δ ppm)	^{1}H NMR δ (nH, m, *J*); ^{13}C NMR δ	Ref
1	-	615.0997 [M-H]$^{-}$; ($C_{28}H_{24}O_{16}$, ΔM -0.8 ppm)	-	-
2	1-*O*-(*E*)-caffeoyl-4,6-di-*O*-galloyl-β-D-glucopyranose	647.1214 [M+H]$^{+}$; ($C_{29}H_{26}O_{17}$ (ΔM 4.4 ppm)	**^{1}H NMR:** *Caffeoyl:* 7.58 (1H, d, 16.0 Hz, H-2'); 7.77 (1H, d, 2.1. Hz, H-4'); 7.55 (1H, dd, 8.3; 2.1 Hz, H-8'); 7.06 (1H, d, 8.3 Hz, H-7');6.28 (1H, d, 16.0 Hz, H-1'); *galloyl*: 6.97 and 6.90 (2H each, s); *glucose*: 5.10 (1H, d, 8.0 Hz, H-1); 4.56 (1H, m, H-4); 4.46 (1H, dd, 11.3, 7.6 Hz, H-6B); 4.33 (1H, dd, 11.3, 7.0 Hz, H-6A); 3.77-3.88 (3H, m, H-2, H-3, H-5)	19
3	Myricetin 3-*O*-α-L-rhamnopyranoside (myricitrin)	463.0880 [M-H]$^{-}$; ($C_{21}H_{20}O_{12}$, ΔM 0.4 ppm)	6.95 (2H, s, H-2'/H-6'); 6.37 (1H, d, 2.2 Hz, H-8); 6.20 (1H, d, 2.2 Hz, H-6); 5.32 (1H, d, 1.5H Hz, H-1"); 4.22 (1H, dd, 3.4, 1.7 Hz, H-2"); 3.79 (1H, dd, 9.6, 3.3 Hz, H-3"); 3.54 (1H, m, H-5"); 3.34 (1H, t, 9.6 Hz, H-4"); 0.94 (3H, d, 6.8 Hz, H-6")	20
4	-	599.1047 [M-H]$^{-}$; ($C_{28}H_{24}O_{15}$, ΔM -0.8 ppm)	-	-
5	Quercetin 3-*O*-(2"-galloyl)-α-L-rhamnopyranoside	599.1041 [M-H]$^{-}$; ($C_{28}H_{24}O_{15}$, ΔM 0.2 ppm)	**^{1}H NMR:** *Quercetin:* 7.34 (1H, d, 2.1 Hz, H-2'); 7.30 (1H, dd, 8.5, 2.1 Hz, H-6'); 6.92 (1H, d, 8.5 Hz, H-5'); 6.38 (1H, d, 2.0 Hz, H-8); 6.21 (1H, d, 2.0 Hz, H-6); *galloyl:* 6.89 (2H, s, H-3'''/H-7'''); *rhamnose:* 5.34 (1H, d, 1.5 Hz, H-1"); 4.78 (1H, dd, 3.4, 1.7 Hz, H-2"); 3.75 (1H, dd, 9.5, 3.4 Hz, H-3"); 3.34 (overlap. signal, H-4"); 3.42 (1H, dd, 9.3, 6.2 Hz, H-5"); 0.94 (3H, d, 6.2 Hz, H-6")	21
6	Mearnsetin 3-*O*-α-L-rhamnopyranoside	477.1041 [M-H]$^{-}$; ($C_{20}H_{22}O_{12}$, ΔM -0.5 ppm)	**^{1}H NMR:** *Mearnsetin:* 6.88 (2H, s, H-2'/H-6'); 6.38 (1H, d, 2.0 Hz, H-8); 6.22 (1H, d, 2.0 Hz, H-6); 3.88 (3H, s, 4-O-CH_3); *rhamnose:* 5.31 (1H, d, 1.7 Hz, H-1"); 4.21 (1H, dd, 3.4, 1.8 Hz, H-2"); 3.74 (1H, dd, 9.5, 3.4 Hz, H-3"); 3.42 (1H, m, H-4"); 3.33 (1H, m, H-5"); 0.95 (3H, d, 6.8 Hz, H-6")	22
7	-	497.1434 [M-H]$^{-}$; ($C_{26}H_{26}O_{10}$, ΔM 3.9 ppm)	-	-
8	Kaempferol 3-*O*-arabinopyranoside	417.0825 [M-H]$^{-}$; $C_{20}H_{18}O_{10}$ (ΔM 0.5 ppm)	**^{1}H NMR:** 8.06 (2H, d, 9.0 Hz, H-2'/H-6'); 6.89 (2H, d, 9.0 Hz; H-3'/H-5'); 6.41 (1H, d, 2.1 Hz, H-8); 6.21 (1H, d, 2.1 Hz, H-6); 5.14 (1H, d, 6.4 Hz, H-1"); 3.60-3.90 (4H, m, H-2"-H-5")	17
9	-	317.0309 [M-H]$^{-}$; $C_{15}H_{10}O_{8}$ (ΔM -1.8 ppm)	-	-
10	-	417.827 [M-H]$^{-}$; $C_{20}H_{18}O_{10}$ (ΔM 0.2 ppm)	-	-
11	-	431.0976 [M-H]$^{-}$; $C_{21}H_{20}O_{10}$ (ΔM 1.8 ppm)	-	-

12	-	459.1294 $[M-H]^-$; $C_{23}H_{24}O_{10}$ (ΔM 0.5 ppm)	-	-
13	Maslinic acid	473.3620 $[M+H]^+$; $C_{30}H_{48}O_4$ (ΔM 0.5 ppm)	**^{1}H NMR:** 5.25 (1H, br. t, 3.6 Hz, H-12); 3.62 (1H, m, H-2); 2.91 (1H, d, 9.5 Hz, H-3); 2.87 (1H, dd, 14.0; 4.1 Hz, H-18); 1.16 (3H, s, CH_3-27); 1.01 (3H, s, CH_3-24); 1.00 (3H, s, CH_3-23); 0.94 (3H, CH_3-30); 0.91 (3H, s, CH_3-29); 0.82 (3H, s, CH_3-26); 0.81 (3H, s, CH_3-25); **^{13}C NMR:** 181.7 (C-28); 145.0 (C-13); 123.1 (C-12); 84.2 (C-3); 69.1 (C-2); 56.4 (C-5); 48.7 (C-9); 47.9 (C-1); 47.2 (C-17); 47.0 (C-19); 42.7 (C-14); 42.5 (C-18); 40.2 (C-8); 40.1 (C-8); 39.0 (C-10); 34.7 (C-21); 33.6 (C-7); 33.4 (C-22); 33.3 (C-29); 31.2 (C-20); 29.0 (C-23); 28.4 (C-15); 26.2 (C-27); 24.1 (C-11); 23.8 (C-30); 23.8 (C-16); 19.1 (C-6); 17.4 (C-26); 17.4 (C-25); 16.9 (C-24)	31
14	3-*epi*-Sumaresinolic acid	473.3625 $[M+H]^+$; $C_{30}H_{48}O_4$ (ΔM 1.7 ppm)	**^{1}H NMR:** 5.28 (1H, br. t, 3.6 Hz, H-12); 4.37 (1H, br s, H-6); 3.28 (1H, t, 2.7 Hz, H-3); 2.87 (1H, dd, 14.0; 4.1 Hz, H-18); 1.31 (3H, s, CH_3-25); 1.22 (3H, s, CH_3-24); 1.15 (3H, s, CH_3-27); 1.09 (3H, s, CH_3-26); 1.00 (3H, s, CH_3-23); 0.94 (3H, CH_3-30); 0.91 (3H, s, CH_3-29); **^{13}C NMR:** 181.8 (C-28); 143.1 (C-13); 123.8 (C-12); 78.1 (C-3); 69.0 (C-6); 56.6 (C-5); 50.0 (C-1); 48.7 (C-9); 47.5 (C-17); 47.1 (C-19); 42.5 (C-18); 42.2 (C-14); 41.0 (C-15); 39.7 (C-8); 38.9 (C-4); 37.1 (C-10); 34.6 (C-21); 33.8 (C-22); 33.3 (C-29); 31.4 (C-20); 28.9 (C-23); 26.3 (C-27);24.6 (C-24); 24.0 (C-11); 23.9 (C-16); 23.7 (C-30); 19.1 (C-2); 18.6 (C-26); 16.9 (C-25)	32
15	Sumaresinolic acid	473.3618 $[M+H]^+$; $C_{30}H_{48}O_4$ (ΔM 1.5 ppm)	**^{1}H NMR:** 5.28 (1H, br. t, 3.5 Hz, H-12); 4.49 (1H, br. s, H-6); 3.07 (1H, dd, 12.0, 4.1 Hz, H-3); 2.87 (1H, dd, 13.9; 3.7 Hz, H-18); 1.30 (3H, s, CH_3-25); 1.15 (3H, s, CH_3-24); 1.12 (3H, s, CH_3-27); 1.09 (3H, s, CH_3-26); 1.04 (3H, s, CH_3-23); 0.94 (3H, CH_3-30); 0.90 (3H, s, CH_3-29); **^{13}C NMR:** 181.3 (C-28); 144.1 (C-13); 123.6 (C-12); 79.8 (C-3); 68.5 (C-6); 56.8 (C-5); 49.0 (C-17); 48.9 (C-9); 46.9 (C-19); 43.1 (C-14); 42.3 (C-18); 41.2 (C-1); 39.7 (C-8); 38.7 (C-4); 38.5 (C-10); 34.6 (C-21); 33.5 (C-22); 33.1 (C-30); 31.3 (C-20); 28.5 (C-15);28.1 (C-24); 27.7 (C-7); 26.1 (C-27); 24.0 (C-11); 23.7 (C-16); 23.5 (C-29); 19.3 (C-2); 18.5 (C-26); 18.5 (C-23); 17.0 (C-25)	32
16	3-*O*-*cis*-*p*-coumaroyl-maslinic acid	619.3979$[M+H]^+$; $C_{39}H_{54}O_6$ (ΔM 2.7 ppm)	**^{1}H NMR:** 7.64 (2H, d, 8.5 Hz, H-2'/6'); 6.88 (1H, d, 12.8 Hz, H-7'); 6.73 (2H, d, 8.5 Hz, H-3'/5'); 5.85 (1H, 12.8 Hz, H-8'); 5.30 (1H, br t, 3.5 Hz, H-12); 4.60 (1H, d, 9.5 Hz, H-3); 3.85 (1H, td, 9.8, 5.7, 1.5 Hz, H-2); 2.88 (1H, dd, 13.6, 4.2 Hz, H-18); 1.16 (3H, s, CH_3-24); 1.13 (3H, s, CH_3-27); 0.94 (3H, s, CH_3-30); 0.90 (9H, overlapping signals, CH_3-23/CH_3-26/CH_3-29); 0.83 (3H, s, CH_3-25); **^{13}C NMR:** 181.7 (C-28); 169.5 (C-9'); 146.0 (C-2'/C-6'); 144.7 (C-13); 144.3 (C-7'); 133.4 (C-1'); 127.3 (C-4'); 123.4 (C-12); 117.0 (C-3'/C-5'); 115.5 (C-8'); 85.3 (C-3); 67.3 (C-2); 57.8 (C-5); 54.1 (C-9); 49.9 (C-17); 47.0 (C-19); 42.9 (C-14); 42.6 (C-18); 42.5 (C-1); 41.3 (C-22); 40.6 (C-4); 40.1 (C-8); 40.0 (C-10); 34.5 (C-21); 33.5 (C-7); 33.2 (C-29); 31.4 (C-20); 30.2 (C-15); 29.0 (C-23); 26.0 (C-27); 24.4 (C-11); 23.8 (C-16); 23.8 (C-30); 19.1 (C-6); 18.7 (C-24); 17.4 (C-25/C-26)	33
17	3-*O*-*trans*-*p*-coumaroyl-maslinic acid	619.3989$[M+H]^+$; $C_{39}H_{54}O_6$ (ΔM 0.7 ppm)	**^{1}H NMR:** 7.63 (1H, d, 15.9 Hz, H-7'); 7.47 (2H, d, 8.6 Hz, H-2'/6'); 6.81 (2H, d, 8.6 Hz, H-3'/5'); 6.39 (1H, 15.9 Hz, H-8'); 5.30 (1H, br t, 3.5 Hz, H-12); 4.64 (1H, d, 10.0 Hz, H-3); 3.84 (1H, td, 10.0, 10.0, 4.0 Hz, H-2); 2.88 (1H, dd, 13.6, 4.2 Hz, H-18); 1.16 (3H, s, CH_3-24); 1.13 (3H, s, CH_3-27); 0.94 (3H, s, CH_3-30); 0.90 (9H, overlapping signals, CH_3-23/CH_3-26/CH_3-29); 0.87 (3H, s, CH_3-25); **^{13}C NMR:** 181.7 (C-28); 169.5 (C-9'); 160.4 (C-4'); 145.9 (C-7'); 144.2 (C-13); 126.9 (C-1'); 130.7 (C-2'/C-6'); 123.4 (C-12); 116.5 (C-3'/C-5'); 115.5 (C-8'); 85.2 (C-3); 67.3 (C-2); 57.7 (C-5); 49.9 (C-17); 48.7 (C-	33

			9); 46.7 (C-19); 43.0 (C-14); 42.5 (C-18); 42.5 (C-8); 42.5 (C-1); 41.0 (C-22); 40.5 (C-4); 39.5 (C-10); 34.5 (C-21); 33.7 (C-7); 33.3 (C-29); 31.3 (C-20); 30.3 (C-15); 28.9 (C-23); 25.9 (C-27); 24.3 (C-11); 23.8 (C-16); 23.7 (C-6); 23.7 (C-30); 18.7 (C-24); 17.4 (C-25); 17.3 (C-26)	
18	3-*O*-trans-*p*-coumaroyl-2α-hydroxydulcioic acid	619.3974 $[M+H]^+$; $C_{39}H_{54}O_6$ (ΔM 3.1 ppm)	**^{1}H NMR:** 7.63 (1H, d, 15.8 Hz, H-7'); 7.46 (2H, d, 9.1 Hz, H-2'/6'); 6.81 (2H, d, 9.1 Hz, H-3'/5'); 6.38 (1H, 15.8 Hz, H-8'); 5.25 (1H, br t, 3.8 Hz, H-12); 4.63 (1H, d, 9.8 Hz, H-3); 3.84 (1H, td, 9.8, 9.8, 4.0 Hz, H-2); 2.88 (1H, dd, 14.4, 4.6 Hz, H-18); 0.97 (3H, s, CH_3-28); 0.95 (3H, d, 6.1 Hz, CH_3-29); 0.95 (3H, s, CH_3-25); 0.94 (3H, s, CH_3-27); 0.90 (3H, s, CH_3-26); 0.90 (3H, s, CH_3-23); 0.87 (3H, s, CH_3-24); **^{13}C NMR:** 181.5 (C-30); 169.3 (C-9'); 160.9 (C-4'); 145.9 (C-7'); 139.5 (C-13); 124.9 (C-1'); 130.7 (C-2'/C-6'); 126.4 (C-12); 116.5 (C-3'/C-5'); 115.4 (C-8'); 85.2 (C-3); 67.4 (C-2); 57.7 (C-5); 49.9 (C-17); 48.4 (C-9); 40.0 (C-19); 40.2 (C-14); 42.7 (C-18); 42.4 (C-8); 48.4 (C-1); 37.8 (C-22); 40.5 (C-4); 40.0 (C-10); 34.5 (C-21); 33.7 (C-7); 23.5 (C-29); 54.0 (C-20); 30.3 (C-15); 28.8 (C-23); 23.5 (C-27); 24.2 (C-11); 23.8 (C-16); 19.2 (C-6); 21.2 (C-28); 17.5 (C-24); 17.8 (C-25); 17.3 (C-26)	34
19	Oleanoic acid	457.3668 $[M+H]^+$; $C_{30}H_{48}O_3$ (ΔM 1.8 ppm)	**^{1}H NMR:** 5.24 (1H, br. t, 3.7 Hz, H-12); 3.14 (1H, dd, 11.6, 4.8 Hz, H-3); 2.85 (1H, dd, 14.3; 4.1 Hz, H-18); 1.16 (3H, s, CH_3-27); 0.97 (3H, s, CH_3-23); 0.94 (3H, s, CH_3-25); 0.94 (3H, s, CH_3-30); 0.90 (3H, s, CH_3-29); 0.82 (3H, CH_3-26); 0.77 (3H, s, CH_3-24); **^{13}C NMR:** 181.4 (C-28); 145.1 (C-13); 123.2 (C-12); 79.3 (C-3); 28.1 (C-2); 56.6 (C-5); 48.7 (C-9); 47.4 (C-17); 46.8 (C-19); 42.6 (C-14); 42.3 (C-18); 39.5 (C-1); 40.1 (C-8); 39.5 (C-4); 37.9 (C-10); 34.6 (C-21); 33.7 (C-22); 33.3 (C-7); 33.2 (C-29); 31.3 (C-20); 27.5 (C-15); 26.0 (C-27); 23.8 (C-11); 23.7 (C-16); 23.7 (C-30); 28.3 (C-23); 18.9 (C-6); 17.2 (C-26); 16.0 (C-24); 15.4 (C-25);	35
20	Ursolic acid	457.3672 $[M+H]^+$; $C_{30}H_{48}O_3$ (ΔM 0.9 ppm)	**^{1}H NMR:** 5.22 (1H, br. t, 3.7 Hz, H-12); 3.15 (1H, dd, 11.6, 4.5 Hz, H-3); 2.20 (1H, d, 11.0 Hz, H-18); 1.11 (3H, s, CH_3-27); 0.97 (3H, s, CH_3-23); 0.96 (3H, d, 6.6 Hz, CH_3-30); 0.95 (3H, s, CH_3-25); 0.88 (3H, d, 6.8 Hz, CH_3-29); 0.85 (3H, CH_3-26); 0.77 (3H, s, CH_3-24); **^{13}C NMR:** 181.6 (C-28); 139.5 (C-13); 126.6 (C-12); 79.4 (C-3); 56.5 (C-5); 54.1 (C-18); 48.8 (C-17); 48.7 (C-9); 40.1 (C-19); 42.9 (C-14); 40.2 (C-1); 40.1 (C-8); 39.6 (C-4); 37.9 (C-10); 34.0 (C-22); 33.3 (C-20); 31.5 (C-21); 31.5 (C-7); 28.9 (C-15); 28.5 (C-23); 27.7 (C-2); 25.0 (C-16); 24.0 (C-11); 23.8 (C-27); 21.3 (C-30); 19.2 (C-6); 17.6 (C-26); 17.4 (C-29); 16.1 (C-24); 15.7 (C-25);	36
